# Examination of the Factor Structure of the Schizotypal Personality Questionnaire among British and Trinidadian Adults

**DOI:** 10.1155/2015/258275

**Published:** 2015-01-28

**Authors:** David Barron, Viren Swami, Tony Towell, Gerard Hutchinson, Kevin D. Morgan

**Affiliations:** ^1^Department of Psychology, Faculty of Science and Technology, University of Westminster, 115 New Cavendish Street, London W1W 6UW, UK; ^2^Department of Psychology, HELP University, BZ-2, Pusat Bandar Damansara (Main Block), 50490 Kuala Lumpur, Malaysia; ^3^Department of Clinical Medical Sciences, University of the West Indies, Mount Hope, Champs Fleurs, Trinidad, Trinidad and Tobago

## Abstract

Much debate in schizotypal research has centred on the factor structure of the Schizotypal Personality Questionnaire (SPQ), with research variously showing higher-order dimensionality consisting of two to seven dimensions. In addition, cross-cultural support for the stability of those factors remains limited. Here, we examined the factor structure of the SPQ among British and Trinidadian adults. Participants from a White British subsample (*n* = 351) resident in the UK and from an African Caribbean subsample (*n* = 284) resident in Trinidad completed the SPQ. The higher-order factor structure of the SPQ was analysed through confirmatory factor analysis, followed by multiple-group analysis for the model of best fit. Between-group differences for sex and ethnicity were investigated using multivariate analysis of variance in relation to the higher-order domains. The model of best-fit was the four-factor structure, which demonstrated measurement invariance across groups. Additionally, these data had an adequate fit for two alternative models: (a) 3-factor and (b) modified 4-factor model. The British subsample had significantly higher scores across all domains than the Trinidadian group, and men scored significantly higher on the *disorganised* domain than women. The four-factor structure received confirmatory support and, importantly, support for use with populations varying in ethnicity and culture.

## 1. Introduction

Despite the debate over the latent structure of schizotypy, studies have revealed a multidimensional structure consisting of at least two factors of positive and negative schizotypy [1]. In parallel with multidimensional schizophrenia models, other suggested schizotypy factors include avoidant symptoms, cognitive disorganisation, social dysfunction, paranoia, and nonconformity [1–6].

The multidimensional structure of schizotypy has been investigated using self-administered scales. One well-established measure that assesses all nine aspects of schizotypal personality disorder in relation to the guidelines of the* Diagnostic and Statistical Manual of Mental Disorders* (5th ed.; DSM-5) is the Schizotypal Personality Questionnaire (SPQ; [7]). These nine schizotypal aspects reflect the following: no close friends, constricted affect, ideas of reference, odd beliefs and magical thinking, unusual perceptual experiences, odd or eccentric behaviour, odd speech, suspiciousness, and excessive social anxiety. First reported by Raine et al. [5] and through subsequent factor analytic studies, these nine subscales can be grouped into three higher-order domains, namely,* cognitive-perceptual*,* interpersonal*, and* disorganised* [2, 8–11].

In terms of factorial structure, Raine et al.'s [5] 3-factor model has been supported, with findings suggesting invariance across age and sex [10, 12] and, in comparison to schizophrenic symptomatology, significant differences in schizotypal traits between sexes [1, 12–16]. In general, women score higher on the* positive* dimension and men score higher on the* negative* and* disorganised* dimensions [1, 12–15].

However, fit indices reported in previous studies have generally been below accepted levels of adequate fit [17–19]. Further, consistency through exploratory [20], principal [17], and confirmatory factor analysis (CFA; [6, 19, 21, 22]) has been problematic. Alternative 3-factor models have also emerged, with Venables and Rector [23] suggesting a model in which* positive schizotypy*,* social avoidance*, and* negative schizotypy* are independent domains.

Other studies have suggested the SPQ may be best suited to a 4-factor structure [6], with researchers utilising this solution over the 3-factor structural model when investigating associations at domain level (e.g., Barron et al. [24]). Stefanis et al. [6] proposed a 4-factor model comprising* cognitive-perceptual*,* paranoid*,* negative*, and* disorganised* dimensions. Confirmation of this structure over alternative solutions has since been obtained [21, 22, 25]. However, with ongoing debate as to the appropriate structure and with few studies only explicitly testing the increased fit in one model compared with an alternative [18], the SPQ factor structure requires further research to clarify its higher-order domains [22].

In addition, despite this research into the SPQ's structure, there has been little work on the dimensions of schizotypy between samples varying in culture and ethnicity, which is important because variability in the dimensionality of the SPQ may limit cross-cultural comparisons. Reynolds et al. [10] found evidence of invariance across ethnicity with the 3-factor structure of the SPQ. Findings with a Mauritian sample provided measurement equivalence between an Indian sample and participants of substantially African origin. Further, there has been evidence of cross-cultural measurement invariance on the Schizotypal Personality Questionnaire-Brief (SPQ-B; [26]) with Swiss and Spanish adolescents [27].

Similarly, despite the translation and wide use of the SPQ and other measures of schizotypal traits, there is a lack of systematic study into the prevalence and manifestation of schizotypy across different cultural and ethnic groups. Cross-cultural research into schizotypy at domain level suggests that African Caribbean populations express greater delusional ideation when compared to White British populations in the UK but not more general schizotypal traits [28]. Chavira et al. [29] examined the relationship between ethnicity and schizotypal personality disorder (SPD), with findings suggesting that African Americans had disproportionally greater SPD diagnoses than Whites and Hispanics. Research on schizophrenia and ethnicity is more exhaustive, with relevant research comparing incidence rates in the Caribbean and UK. The incidence of schizophrenia in Jamaica [30], Trinidad [31], and Barbados [32] has been found to be similar to the rate for the White population in England, which contrasts with the elevated incidence of schizophrenia in African Caribbean populations in UK [33]. As healthy individuals who express schizotypal traits have a higher risk of developing schizophrenia-spectrum disorders [34–37], an investigation of schizotypy in African Caribbean and White British samples could further illuminate not only the nature of schizotypy as a personality dimension but also its link to variations of schizophrenia risk in different ethnic groups.

Using CFA, therefore, we sought to identify the model of best fit between the 3-factor [5] and 4-factor structures [6]. While this is not an exhaustive account of possible structures, these represent the two most common solutions in the literature. Following Compton et al. [22], three hierarchically related models were also investigated based on the 4-factor structure. Second, we examined the measurement equivalence of the best fitting model of the SPQ across White British participants in London, UK, and African Caribbean participants in Port of Spain, Trinidad and Tobago. Finally, we compared domain-level scores across cultural groups and sex.

## 2. Method

### 2.1. Participants

There were 635 participants: 351 (55.3%) White British residents in London, UK, and 284 (44.7%) African Caribbean residents in Port of Spain, Trinidad. Both subsamples comprised participants from the general public and undergraduates. Recruitment from the general public was primarily through recruitment agencies in Trinidad and social and religious groups in London and Trinidad. The mean age of participants was 26.06 (SD = 9.83) years for the British subsample with 226 (64.4%) women and 125 (35.6%) men and 28.79 (SD = 7.70) years for the African Caribbean subsample with 204 (71.8%) women and 80 (28.2%) men. The African Caribbean subsample was significantly older, *t*(632) = 3.82, *P* < .001, and *d* = 0.31, than the British subsample. All participants self-reported as not having a history of mental health problems relating to psychosis.

### 2.2. Measures

**Figure 1:**
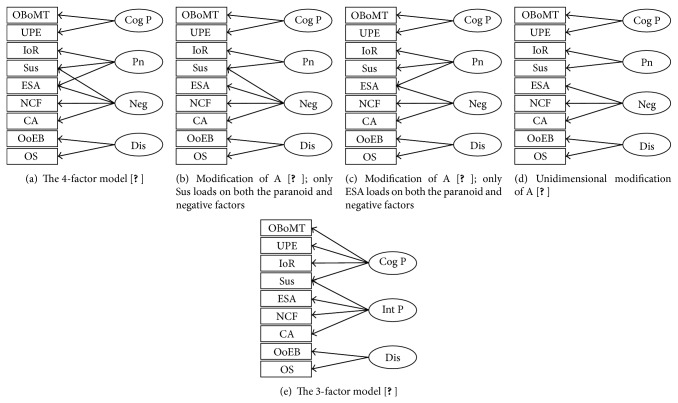
The measurement models under examination. Factors: Cog P:* cognitive-perceptual*, Pn:* paranoid*, Neg:* negative*, Dis:* disorganised*, and Int P: interpersonal. Subscales: OBoMT: odd beliefs or magical thinking, UPE: unusual perceptual experiences, IoR: ideas of reference, Sus: suspiciousness, ESA: excessive social anxiety, NCF: no close friends, CA: constricted affect, OoEB: odd or eccentric behaviour, and OS: odd speech.

Participants completed the 74-item Schizotypal Personality Questionnaire (SPQ; [7]), designed to measure all nine diagnostic criteria for schizotypal personality disorder. Each “yes” response counts as one point and 9 subscale scores were computed as the total score for all items associated with each subscale.  Table 1 shows Cronbach's alpha coefficients for the 9 subscales in the present sample (range = .70–.83, mean = .77), which is in-line previous findings [22]. Domain scores were derived by summing of relevant subscale scores (see Figure 1).

### 2.3. Procedure

Ethics approval for this study was obtained from the relevant university ethics committee. Survey dissemination was undertaken via multiple routes. First, an internal online research participation scheme was utilised. This scheme gives course credit to students eligible for this incentive. No monetary incentives were offered to the participants for completion of the survey. Second, individuals were invited to participate via a paper-and-pencil format. In both the offline and online versions, participants completed a consent form before proceeding to the survey. Further, study information was distributed to the general public through recruitment agencies and social and religious groups in both London and Trinidad. All participants received written debrief information at the end of the study.

### 2.4. Data Analysis

CFAs were conducted using Analysis of Moment Structures (AMOS 21; [38]) to examine the factorial structure of the SPQ. Two prominent models proposed in the literature are the 3-factor [5] and 4-factor [6] models. These were examined with three further hierarchically related models (see Figure 1), which have previously been investigated [22]. Standard goodness-of-fit indices were selected* a priori* to assess the measurement models. The normed model chi-square (*χ*_normed_^2^) is reported with lower values of the overall model chi-square indicating goodness-of-fit (<3.00 indicates good fit). The Steiger-Lind root mean square error of approximation (RMSEA) and its 90% confidence interval provide a correction for model complexity (<0.08 indicates good fit). The standardized root mean square residual (SRMR) assesses the mean absolute correlation residual. The smaller the SRMR, the better the model fit (<0.80 indicates good fit). The comparative fit index (CFI) measures the proportionate improvement in fit by comparing a target model with a more restricted, nested baseline model.Generally, the CFI is recommended to be >0.90. The Akaike information criterion (AIC) provides a measure to compare nonhierarchical factor structures, with the lowest AIC value being preferred. To compare hierarchical models, the chi-square difference test was used. A nonsignificant value indicates an equal fit when comparing the models. This allows testing for the invariance of the models across ethnicity by way of a multiple-group analysis. A multivariate analysis of covariance (MANCOVA) was used to examine sex and ethnicity differences with the domains structure for the model of best fit, with participant age entered as a covariate term. The Bonferroni correction was used for multiple comparisons.

## 3. Results

### 3.1. Confirmatory Factor Analysis

The goodness-of-fit indices of the five models proposed are shown in Table 2. As depicted, the first model (A) is the multidimensional 4-factor model of Stefanis et al. [6], which fits the data well. Previous research [22] has investigated two multidimensional modifications of this well-fitting model, one (B) where the suspiciousness subscale, but not excessive social anxiety, loads on the* paranoid* and* negative* dimensions and the other (C) in which excessive social anxiety, but not suspiciousness, loads on the* paranoid* and* negative* dimensions, and one unidimensional modification (D) in which indicators do not load on more than one dimension. With the exception of (B), previous research into these modifications has indicated a relatively poor fit [22]. Similarly, the first modification (B) had good fit with alternative modifications having poor fit. The final model (E) is Raine et al.'s [5] multidimensional 3-factor model. This model fits the data adequately.

Overall, three of the models examined fit the data adequately, with all indices being within acceptable ranges. The modified 4-factor structure (B) had a better fit than the 3-factor Raine et al. [5] model but poorer fit than the Stefanis et al. [6] model. Further, using the AIC of the models as a comparative measure of fit, the Stefanis et al. [6] model had the best fit. Therefore, from the three well-fitting models, the present data were best suited to the Stefanis et al. [6] 4-factor model.

To test for invariance of the models across ethnicity, we performed multiple-group analyses with the best fitting and two well-fitting models. As the unconstrained model fits each sample individually in each model, we compared the constrained and unconstrained *χ* ^2^ and respective df values. The differences between the *χ* ^2^ and df values were not significant, indicating that the structure of the model was invariant across groups and this was consistent in each model.

### 3.2. Between-Group Differences

We further investigated sex and ethnicity differences in scoring with the four domains of the best fitting model. A 2-way MANCOVA was conducted, with the four dimension scores as dependent variables and age entered as a covariate term. A statistically significant main effect was obtained for ethnicity, *F*(4,626) = 44.60, *P* < .001, Wilk's Λ = .78, and *η*_*p*_^2^ = .22. A series of follow-up one-way analyses of variance (ANOVAs) indicated that, for each domain, including the total schizotypy score, the British subsample scored significantly higher than the African Caribbean subsample (see Table 3). There was a significant main effect of sex, *F*(4,626) = 5.79, *P* < .01, and Wilk's Λ = .96, *η*_*p*_^2^ = .04. Further, one-way ANOVAs indicated a significant difference in the* disorganised* domain only, with men (*M* = 4.90, SD = 4.23) scoring significantly higher than women (*M* = 3.66, SD = 3.83), *F*(1,629) = 7.89, *P* < .01, and *η*_*p*_^2^ = .01. There was a significant ethnicity × sex interaction, *F*(4,626) = 3.54, *P* < .01, and *η*_*p*_^2^ = .02. However, the effect size of the interaction was small and inspection of the one-way ANOVAs indicated that none of the effects reached significance.

## 4. Discussion

The present findings revealed that the original 3-factor structure [5], the 4-factor [6], and a hierarchically related 4-factor structure [22] had fit indices within an acceptable range. Of these well-fitting models, the 4-factor model proposed by Stefanis et al. [6] had the best fit. Raine et al.'s [5] 3-factor model fitted well, which is consistent with some previous investigations into the SPQ's structure [10]. However, the 3-factor solution did not fit as well as the two 4-factor structures, indicating that the presence of a* paranoid* factor may improve fit. Further, the modification whereby suspiciousness, but not excessive social anxiety, loaded on both the* paranoid* and* negative* factors had the second best fit, which is consistent with previous findings [22].

In addition, measurement invariance was found for the three well-fitting models. These findings suggest confidence in the factorial structure and robustness between the divergent samples. As measurement invariance was obtained, it may be assumed that change in the latent mean score reflects the latent variable and not an artefact of the measurement tool. This supports previous evidence of measurement invariance for the 3-factor model [10] and 3- and 4-factor models of the SPQ-B [14], with the addition of support for the 4-factor structure of the SPQ.

Subsequent analyses for each domain, derived from the best fitting four-factor model, revealed that men scored higher on the* disorganised* factor than women. While this is a consistent finding, previous research has found that men score higher on the* negative* factor and women score higher on the* cognitive-perceptual* factor [1, 13, 15, 19], which were not established in the present study. The* cognitive-perceptual* and* negative* domains may be influenced by the inclusion of the* paranoid* domain in the 4-factor solution, in terms of the lower-order to higher-order structure. While not being in the scope of the present research, it would be of interest to further investigate this effect related to respondent sex. With regard to the* cognitive-perceptual* factor, reducing the four lower-order factors in the 3-factor solution to two lower-order factors in the 4-factor model may have diminished the effect of sex with this domain and should be further examined.

Finally, between-group analysis of the subgroups indicated that the White British group scored significantly higher than the African Caribbean group on the four higher-order domains. When considering the schizophrenia literature, similar incidence rates between White British and African Caribbeans have been found when the sample has been recruited from UK and Trinidad, respectively [31]. While the difference between the subgroups may reveal the profile of schizotypy to be dissimilar to that of schizophrenia, it could possibly be explained by confounding variables, such as urbanicity. Research suggests that around one-third of all diagnoses of schizophrenia may be associated with environmental factors related to the urban environment [39]. With the diverse sociodemographic variables of the subsamples particularly in relation to population density and socioeconomic status, the urban environment, rather than ethnicity, may account for variation in domain scores.

Further, methodological problems may account for some of the difference in schizotypal scores. This study adopted both paper-and-pencil and electronic versions of the SPQ. While the majority of the UK subsample completed the SPQ online, the Trinidadian subsample required a paper-and-pencil approach. Buchanan et al. [40] reported nonequivalence between online and paper-and-pencil approaches, suggesting caution with the measurement of psychological properties online. However, with a lack of internet access for many in Trinidad, this subsample was restricted primarily to a paper-and-pencil format of the SPQ. With online personality measurement, there is a lack of control in testing and the possibility of extraneous (e.g., environmental cues) or temporary (e.g., fatigue) factors influencing respondents [41]. Further, in the present research, factors such as language and cultural differences may also be important, as well as interactions between the measured constructs and the characteristics of the testing method [41]. With a lack of literature relating to the testing medium of the SPQ, it is unclear whether respondents would be influenced by either method.

Thus, future research should investigate the best-suited testing medium for the SPQ and continue to investigate the SPQ's structure and measurement invariance across ethnicity and culture. In particular, expanding upon the present study with the addition of a culture-controlled comparison, such as a UK-based African Caribbean subsample, would be a useful direction for further research. Further refinement of the structure and knowledge regarding the SPQ will advance this assessment tool, allowing it to be used in community studies and in parallel with endophenotypes for the early detection of schizophrenia.

## Figures and Tables

**Table 1:** Cronbach's alpha for SPQ subscales.

SPQ subscale	α
Ideas of reference (IoR)	.78
Excessive social anxiety (ESA)	.83
Odd beliefs or magical thinking (OBoMT)	.73
Unusual perceptual experiences (UPE)	.74
Odd or eccentric behaviour (OoEB)	.83
No close friends (NCF)	.79
Odd speech (OS)	.73
Constricted affect (CA)	.70
Suspiciousness (Sus)	.78

*P* < .001;  *P* < 0.01.

**Table 2:** Indices for each proposed model.

Model		*χ*_*M*_^2^	df_*M*_	*χ*_normed_^2^	RMSEA (90% CI)	SRMR	CFI	AIC
A.	The 4-factor model [6]	74.12	36	2.06	.041 (.028, .054)	.025	.99	182.12

B.	Modification of A [22] Only Sus loads on both the paranoid and negative factors	79.77	38	2.10	.042 (.029, .054)	.029	.98	183.77

C.	Modification of A [22] Only ESA loads on both the paranoid and negative factors	118.27	38	3.11	.058 (.046, .070)	.039	.97	222.27

D.	Unidimensional modification of A [22]	123.18	40	3.08	.057 (.046, .069)	.042	.97	223.18

E.	The 3-factor model [5]	83.73	38	2.20	.044 (.031, .056)	.029	.98	187.73

**Table 3:** Ethnicity ratings within the best fitting model.

Domains	African Caribbean mean (SD)	White British mean (SD)
*Cognitive-perceptual* ^*^	1.87 (2.36)	3.18 (3.27)
*Paranoid* ^**^	7.13 (5.13)	8.67 (6.04)
*Negative* ^*^	7.12 (5.88)	10.26 (7.39)
*Disorganised* ^*^	2.14 (2.83)	5.61 (4.14)

Total^*^	13.74 (10.77)	21.54 (14.26)

^*^ *P* < .001;  ^**^ *P* < .05.
